# On the Use of Cinnamon in Certain Examples of Menorrhagia

**Published:** 1853-12

**Authors:** T. H. Tanner

**Affiliations:** Licentiate of the Royal College of Physicians; Physician to the Hospital for Women, &c.


*On the Use of Cinnamon in Certain Examples of Menorrhagia*—By T. H. Tanner, M. D.—Licentiate of the Royal College of Physicians; Physician to the Hospital for Women, &c.—Amongst the numerous cases of menorrhagia which come under my notice in hospital and private practice, instances are not unfrequently met with where no satisfactory explanation can be given for the increased catamenial flow. Of course, amongst these, patients suffering from general plethora, from hæmorrhage caused by polypi either within or without the uterus, or from that dependent upon fibrous tumours, uterine hydatids, ulceration (simple or malignant), or upon a watery condition of the blood, are not included. In the instances alluded to, I have almost always been unable, after careful examination, to say upon what the menorrhagia depended, since no physical alteration could be detected in either the uterus or ovaries; it has only therefore been practicable to assert what were *not* the causes.

The syptoms usually presented are briefly these: the catamenia appear regularly every twenty eight days, and are at first only of the proper quantity; but, instead of ceasing after a duration of three or four days, they continue unabated for ten or fourteen, and occasionally even for three weeks. The general symptoms which arise from this debilitating discharge are just as might be expected. There is general weakness, langour, mental depression, with pains in the head, loins, and so on; the patient suffering, it is to be remembered, not from any diseased condition, giving rise to the hæmorrhage, but merely from the loss of blood itself. In other instances the discharge continues a less time, but the flow is more abundant, clots being frequently discharged; this variety is generally followed by leucorrhœa. My own experience tends to show that these forms of menorrhagia occur more frequently in unmarried than in married women; but, for many reasons, and especially because of the class of patients from whom my observations are for the most part deduced, I would not say positively that such is the case.

Ailments of this class are at all times troublesome to cure, but those I am considering are particularly so, from the absence of any special indications for treatment. In some of them, indeed, it appears, at first sight, only necessary to keep the patient quiet, to administer astringents, especially such as act particularly upon the uterus, and to regulate the diet, in order to give the desired relief. But it will often be found that these means are quite inefficient, and that the acetate of lead, gallic acid, the ergot of rye, oxide of silver, sulphuric acid, tincture of sesquichloride of iron, and similar remedies may be employed without any avail. In thinking over these cases I was led, from a remark made by Dr. Pereira, to try the use of cinnamon. In the last edition of the "Elements of Materia Medica," this gentleman says, when speaking of cinnamon, that "some writers regard it as acting specifically on the uterus," (vol ii. p. 1307,) and reference is then made to the writings of Sundelin and Wibmer. As far as I know, these are the principal authors who have recommended the use of this agent; for I am not aware that mention is made of it by gentlemen who have written on obstetrics and the diseases of women in this country. Having thus been led to try the effects of this agent, and having derived the most beneficial effects from its employment, I have felt desirous of making its value more generally known, that its utility may be tested on a large scale.

That its beneficial action is really due to some specific effect which cinnamon exercises upon the uterus, and not any astringent property it may possess from the tannic acid which the bark and leaves of it contain in common with all the lauraceæ order of plants, is, I think, certain; and partly in confirmation of this view, it may be mentioned, that in a case of labor in which I employed it, it *appeared* not only in a marked manner to increase the severity and rapidity of the pains, but the patient, who had in her previous labours suffered severely from flooding after the birth of the placenta, on this occasion lost only a very small quantity of blood. That this fortunate circumstance was due to the administration of the cinnamon I do not of course pretend positively to assert; the case is merely mentioned to give some color to the opinion expressed that this agent acts specifically upon the uterus. Much clearer evidence of its value in such cases of menorrhagia as I have described, is, however, easily obtained. The principal points I would now refer to are, that it acts after the failure of other astringents; that it is most efficacious given alone, uncombined with other medicines; that if its employment be discontinued too soon, the discharge of blood returns ; while, in a few instances it has been found necessary, after an apparent cure, to resort to its employment for the two succeeding catamenial periods, when the menstrual flow after continuing for three or four days, has not given any signs of abatement, and when the patient has begun to suffer from mental depression and the early symptoms of general debility. The form usually employed, and which appears to be the best, is that of the tincture, in drachm doses, using cinnamon water as the vehicle ; it should be taken about every six hours, but not more frequently, as it is apt to give rise to nausea and vomiting. It is also better to continue its administration for about fourteen days after the symptoms which called for its employment have disappeared ; and even then, if the case has been an obstinate one, a draught composed of it should be taken once daily for a month.

In concluding these brief observations, I trust I shall not be understood as recommending cinnamon as an infallible remedy in all examples of menorrhagia. All I would wish to assert is, that in a certain class of cases I have found it to possess properties of great value ; and though I might have waited until I had been enabled to test its properties on a larger scale, yet I preferred giving the results of my experience at once, in order that the readers of THE LANCET might have the opportunity of employing this agent, and ascertaining its value for themselves. If they will do so, and record the result, we shall soon possess satisfactory data for determining the action of cinnamon upon the uterus.—*London Lancet.*

